# Genome-wide analysis of RWP-RK transcription factor family reveals its roles in nitrogen response in rice (*Oryza sativa*)

**DOI:** 10.3389/fpls.2025.1597029

**Published:** 2025-09-08

**Authors:** Mei Du, Ziwen Guo, Siyu Gong, Liangwei Xu

**Affiliations:** ^1^ Sanya Nanfan Research Institute, Hainan University, Sanya, Hainan, China; ^2^ School of Tropical Agriculture and Forestry, Hainan University, Haikou, Hainan, China; ^3^ Rice Innovation Team, Yazhouwan National Laboratory, Sanya, Hainan, China; ^4^ Hainan Seed Innovation Research Institute, Sanya, Hainan, China

**Keywords:** RWP-RK, NLP proteins, RKD proteins, transcription factors, nitrogen starvation, rice

## Abstract

**Introduction:**

The RWP-RK transcription factor family plays a pivotal role in nitrogen response and gametophyte development. Despite its biological importance, the evolutionary relationships and functional characteristics of RWP-RK genes in rice remain incompletely understood. This study aimed to investigate the structure, classification, expression patterns, and potential regulatory roles of RWP-RK transcription factors in rice (Oryza sativa), with a focus on their involvement in nitrogen signaling and reproductive development.

**Methods:**

A comprehensive genome-wide analysis was conducted to identify RWP-RK genes in rice. A total of 13 genes encoding 14 proteins, including two alternative splicing variants of OsNLP5, were identified and mapped across 8 of the 12 rice chromosomes. Phylogenetic analysis was used to classify the proteins into subfamilies, and gene/protein structure characteristics were examined, including coding sequence length, exon number, and domain composition. Collinearity analysis was performed to explore evolutionary relationships between rice and *Arabidopsis*. Promoter regions of the RWP-RK genes were analyzed for cis-regulatory elements, and tissue-specific as well as nitrogen-responsive gene expression patterns were evaluated using expression profiling.

**Results:**

Phylogenetic analysis grouped the 14 RWP-RK proteins into four clades: groups 1–3 were assigned to the NLP subfamily and group 4 to the RKD subfamily. NLP members contained both RWP-RK and PB1 domains, while RKD proteins possessed only the RWP-RK domain. Structural analysis revealed that NLP genes generally have longer CDS, more exons, and larger proteins than RKD genes. Collinearity analysis suggested that rice and *Arabidopsis* RWP-RK genes share a common ancestor, with evidence of gene recombination and species-specific divergence. Promoter analysis revealed numerous hormone- and stress-responsive cis-elements. Expression profiling showed that OsNLP genes are broadly expressed in all tissues, whereas OsRKD genes are predominantly active in reproductive organs. Upon nitrogen resupply after nitrogen starvation, expression levels of all OsNLP genes and three OsRKDs (OsRKD1, OsRKD3, OsRKD5) showed dynamic changes.

**Discussion:**

The findings provide new insights into the classification, structure, and expression dynamics of the RWP-RK transcription factor family in rice. The distinct domain architectures and expression patterns between the NLP and RKD subfamilies suggest functional divergence, with NLP genes potentially playing broader roles in general nitrogen regulation, while RKD genes may be more specialized for reproductive development. The nitrogen-responsive expression changes highlight the potential regulatory role of these transcription factors in nutrient signaling. Overall, this study lays a valuable foundation for future functional investigations into the OsRWP-RK family's roles in nitrogen response and gametophyte development in rice.

## 1 Introduction

Nitrogen is an essential plant macronutrient, constituting a fundamental component of proteins, nucleic acids, and chlorophyll. Soil nitrogen mainly originates from microbial nitrogen fixation and anthropogenic input (Li et al., 2024). Its deficiency severely impairs plant growth and development. In rice, nitrogen shortage reduces biomass, tiller number, and ultimately affects yield and quality (Hong et al., 2022). However, plant roots absorb only 30% of the available soil nitrogen, with the remaining 70% lost through leaching or gaseous emissions, causing environmental degradation, such as eutrophication and the greenhouse effect (Zhang et al., 2024). Therefore, developing crop varieties that efficiently acquire and utilize nitrogen, while maintaining high yields under reduced nitrogen input is a sustainable solution (Tegeder and Masclaux-Daubresse, 2018). Transcription factors containing the RWP-RK domain play a crucial role in regulating genes involved in nitrogen signaling and metabolism, which are vital for plant growth and development (Yan and Nambara, 2023).

RWP-RK transcription factors contain a conserved 60-amino acid RWP-RK motif, an ancient motif predating the emergence of Viridiplantae (green algae and land plants) (Sakuraba et al., 2022). In the process of evolution, they are classified into two subfamilies—NIN-like proteins (NLPs) and RWP-RK domain proteins (RKDs)—based on protein sequence (Chardin et al., 2014). NLPs feature three characteristic domains: an RWP-RK domain that binds cis-acting elements in NUE-related gene promoters (Konishi and Yanagisawa, 2013), a PB1 domain for protein-protein interactions (Konishi and Yanagisawa, 2019), and an N-terminal GAF-like domain (widely present in animals, bacteria and fungi, but is absent in plants) potentially involved in signal transduction (Chardin et al., 2014; Liu et al., 2017). RKDs, on the other hand, contain a conserved RWP-RK domain, regulating genes involved in gametogenesis and embryogenesis (Koszegi et al., 2011).

NLPs are involved in the transcriptional activation of key genes related to nitrogen uptake, assimilation, and nitrate signaling, ensuring efficient nitrogen for plants under nutrient-limited conditions (Yan and Nambara, 2023). A mutant of seven *Arabidopsis* NLP transcription factors (*nlp2, 4, 5, 6, 7, 8, 9*) impairs primary nitrate responses and developmental processes of seedlings (Liu et al., 2022). The function of AtNLP7 has been extensively studied, it acts as both a transcription activator and an intracellular nitrate sensor, regulated by nitrate through a nuclear retention mechanism. AtNLP7 binds and modulates most known nitrate signaling and assimilation genes (Marchive et al., 2013; Liu et al., 2022; Durand et al., 2023). AtNLP2, AtNLP6, and AtNLP8 also play vital roles in nitrate signaling, with AtNLP2 regulating early nitrate responses and linking nitrate assimilation to energy and carbon supply (Durand et al., 2023), AtNLP6 activating nitrate signaling and supporting root meristem growth (Guan et al., 2017), and AtNLP8 promoting nitrate-dependent seed germination (Yan et al., 2016). In rice, OsNLP3, the homolog of AtNLP7, together with OsNLP1 and OsNLP4, directly binds to the promoters of nitrate-responsive genes (Hu et al., 2019; Alfatih et al., 2020; Wang et al., 2021; Wu et al., 2021; Zhang et al., 2022), playing overlapping yet distinct roles in nitrogen acquisition, assimilation, and nitrogen use efficiency (NUE), thereby enhancing grain yield (Yan and Nambara, 2023). OsNLP3 and OsNLP4 primarily govern nitrate metabolism, with mutants showing significant growth inhibition and decreased NUE under nitrate conditions, but no notable differences under ammonium conditions (Wang et al., 2021; Wu et al., 2021; Zhang et al., 2022). In contrast, OsNLP1 regulates both nitrate and ammonium metabolism, coordinating the expression of relevant genes and conferring broad adaptability to these nitrogen sources (Alfatih et al., 2020). OsNLP3 and OsNLP1 transcripts are both induced by nitrogen starvation, with OsNLP1 protein localizing in the nucleus, while OsNLP3 nucleo-cytosolic shuttling is specifically regulated by nitrate (Alfatih et al., 2020; Zhang et al., 2022). OsNLP2 localizes to the nucleus and negatively regulates ferroptotic cell death and immune responses during *Magnaporthe oryzae* infection [20]. Additionally, NLP2-NR-associated NO enhances salt stress tolerance by promoting ABA catabolism during seed germination (Yi et al., 2022).

The RKD (RWP-RK domain-containing) transcription factors possess a highly conserved RWP-RK domain (Koszegi et al., 2011). Recent studies have demonstrated that RKD transcription factors are crucial in germ cell differentiation and embryo development in land plants. In *Arabidopsis*, AtRKD1 and AtRKD2 are predominantly expressed in egg cells; their ectopic expression promotes cell proliferation and activates an egg cell marker (Koszegi et al., 2011). AtRKD3, an ovule development-specific gene, regulates germ cell differentiation and embryo development through transcriptional activation or repression of target genes (Tedeschi et al., 2017). AtRKD4 is preferentially expressed in early embryos and activates early embryo-specific genes (Waki et al., 2011). Moreover, in rice, OsRKD3 induces somatic embryo formation in the normally somatic embryogenesis-resistant Indonesian black rice landrace (Cempo Ireng) (Purwestri et al., 2023).

The RWP-RK transcription factor involves various physiological processes, such as nitrogen response, gametophyte development, and abiotic stress regulation (Chardin et al., 2014). In many plants, including wheat (Kumar et al., 2018), maize (Ge et al., 2018), brassica (Liu et al., 2018), and soybean (Amin et al., 2023), genome-wide identification and characterization of the RWP-RK family have been reported. RWP-RK transcription factors are crucial for rice nitrogen utilization and embryo development, but genome-wide analyses of rice RWP-RKs are scarce. Leif Schauser’s study on OsNLP1, OsNLP2, and OsNLP3 revealed that NLPs play a key role in rice nitrogen signaling (Schauser et al., 2005). In 2020, B. Jagadhesan investigated OsNLP1-6 in four rice lines (APO, IR83929-B-B-291-3-1-1, Nerica-L-42, and Pusa Basmati 1) and identified OsNLP1 and OsNLP3 as major contributors to nitrogen use efficiency (Jagadhesan et al., 2020). However, these studies focus mainly on individual genes or genotypes, lacking comprehensive genome-wide identification and expression analyses. In this study, 13 OsRWP-RK genes (14 proteins, including two proteins of OsNLP5) were identified, their conserved motifs, gene structure, protein structures, and promoter elements were analyzed. The expression patterns of OsRWP-RK genes were examined using transcriptome data. Additionally, expression changes in roots were assessed after transferring nitrogen-deprived rice seedlings to nitrogen-sufficient conditions. This study provides a deeper understanding of RWP-RK genes in rice. It lays a theoretical foundation for further research on the nitrogen signaling pathway and gametophyte development.

## 2 Materials and methods

### 2.1 Identification of RWP-RK transcription factors family members in rice

To identify RWP-RK domain-containing transcription factors in rice (*Oryza sativa*), the rice protein database was obtained from JGI (https://phytozome-next.jgi.doe.gov; accessed on 5 Jan 2025; *Osativa* 323 v7.0) (Goodstein et al., 2012). Raw Hidden Markov Model (HMM) data of the conserved RWP-RK (PF02042) domain were downloaded from the Pfam database (http://pfam.xfam.org; accessed on 5 Jan 2025) and used to search for orthologues in the rice genome (Mistry et al., 2021). The RWP-RK domain in candidate proteins was verified using the NCBI Conserved Domains search tool (https://www.ncbi.nlm.nih.gov/Structure/bwrpsb/bwrpsb.cgi; accessed on 5 Jan 2025) (Marchler-Bauer et al., 2017; Wang et al., 2023). The physicochemical properties of OsRWP-RK proteins were predicted using ProtParam (https://web.expasy.org/protparam/; accessed on 5 Jan 2025) (Wilkins et al., 1999), and their subcellular localizations were predicted using Cell-PLoc (http://www.csbio.sjtu.edu.cn/bioinf/plant-multi/; accessed on 5 Jan 2025) (Chou and Shen, 2010).

### 2.2 Phylogenetic tree, chromosomal distribution, and synteny analysis

*Arabidopsis* RWP-RK protein sequences were obtained from PlantTFDB v5.0 (https://planttfdb.gao-lab.org; accessed on 3 Jan 2025) (Jin et al., 2017). Sequences of 28 RWP-RK proteins from *Arabidopsis thaliana* and *Oryza sativa* were aligned using Mafft, and a phylogenetic tree was constructed with the maximum likelihood (ML) method in Fasttree, including bootstrap analysis with 500 replicates. Chromosomal localization and synteny analysis were performed using TBtools (version 2.194) (Chen et al., 2023).

### 2.3 Gene structures, conserved structural domains, conserved motifs

Gene structure data were obtained from the genome annotation file on JGI (https://phytozome-next.jgi.doe.gov; accessed on 5 Jan 2025; *Osativa* 323 v7.0) and analyzed using TBtools (version 2.194) (Goodstein et al., 2012; Chen et al., 2023). Conserved motifs of OsRWP-RK proteins were identified with MEME (http://meme-suite.org/tools/meme; accessed on 3 Jan 2025) (Bailey et al., 2015), and the top three motifs were extracted. Conserved domains were predicted using NCBI-CDD (http://www.ncbi.nlm.nih.gov/Structure/cdd/wrpsb.cgi; accessed on 3 Jan 2025) and visualized in TBtools (version 2.194) (Marchler-Bauer et al., 2017; Chen et al., 2023; Wang et al., 2023).

### 2.4 Protein 3D structure prediction

The 3D structure of the OsRWP-RK proteins was predicted using the AlphaFold Protein Structure Database (https://alphafold.com/; accessed on 14 Jan 2025) (Jumper et al., 2021; Varadi et al., 2023).

### 2.5 OsRWP-RK gene promoter analysis

To analyze the promoter sequences of OsRWP-RK transcription factors, 2000 bp upstream of each gene’s initiation codon was extracted using TBtools (version 2.194) (Chen et al., 2023). Cis-elements were predicted with PlantCARE (http://bioinformatics.psb.ugent.be/webtools/plantcare/html; accessed on 6 Jan 2025) and visualized using TBtools (version 2.194) (Lescot et al., 2002; Chen et al., 2023).

### 2.6 Transcription profile of OsRWP-RK genes

Expression data for five rice tissues (leaf, root, stem, inflorescence, and seed) were obtained from the Rice Gene Annotation Project (https://rice.uga.edu/expression.shtml; accessed on 7 Jan 2025). Heatmaps and histograms were generated using GraphPad Prism (version 9.5).

### 2.7 Nitrogen induces treatments, RNA extraction, and qRT-PCR analysis

Rice seedlings of the Japonica variety ZH11 were grown for 15 days in a modified Kimura B nutrient solution (1 mM NO_3_^-^ + 1 mM NH_4_^+^) and then transferred to a nitrogen-free solution for 5 days. After nitrogen starvation, plants were returned to the modified solution, and roots were collected at 0, 10, 30, 60, and 120 minutes to obtain RNA. RNA was extracted using RNA isolator Total RNA Extraction Reagent (R401-01, Vazyme) and reverse transcribed with HiScript III All-in-one RT SuperMix (R333, Vazyme). Quantitative PCR was performed using ChamQ Universal SYBR qPCR Master Mix (Q711-02, Vazyme) on a LightCycler^®^ 480 Instrument II. The primers used for qRT-PCR are listed in **Supplementary Data Sheet S1**. The experiment was conducted with three biological replicates.

## 3 Result

### 3.1 Identification and classification of OsRWP-RK genes

**Figure 1:**
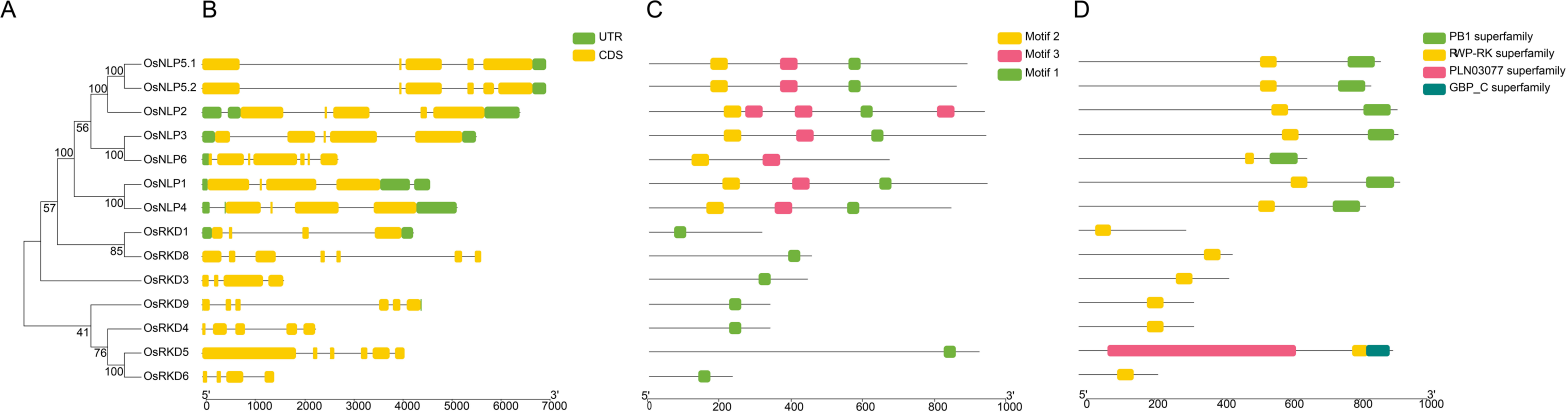
Phylogenetic analysis, gene structure, and conserved motifs and domain of OsRWP-RK proteins in *Oryza sativa*. **(A)** Phylogenetic tree of 14 OsRWP-RK proteins. **(B)** Gene structure showing exons (yellow bars), introns (black lines), and upstream/downstream regions (green bars). **(C)** Conserved motif distribution, with each motif represented by a distinct color. **(D)** Conserved domain organization, with different domains highlighted in colored boxes.

The *Oryza sativa* genome contains 13 RWP-RK genes encoding 14 protein isoforms, including two variants of OsNLP5 (**Table 1**), all harboring the conserved RWP-RK domain (**Figure 1**). The coding sequence (CDS) lengths of the OsRWP-RK family range from 699 bp (OsRKD6) to 2829 bp (OsNLP1), with corresponding protein lengths spanning 232 (OsRKD6) to 942 amino acids (OsNLP1). Their molecular weights vary from 27.4 kDa (OsRKD6) to 104.7 kDa (OsRKD5), and predicted protein isoelectric points range from 5.17 (OsNLP6) to 8.46 (OsRKD4) (**Table 1**). Proteins in the NLP subfamily generally possess longer CDS and higher molecular weights compared to those in the RKD subfamily (**Table 1**, **Supplementary Figure 1**), whereas OsRKD5 exhibits similar features to the NLP subfamily (**Table 1**).

**Table 1:** The characteristics of putative RWP-RK protein genes in *Oryza sativa subsp. Japonica*.

Gene name	Gene ID_MSU	Chr	Start	End	CDS (coding sequences) length in bp	Length of protein in AA (amino acid)	MW (molecular weights) (KDa)	PI (protein isoelectric points)	Group	Predicted location	Gene product name
OsNLP1	LOC_Os03g03900	Chr3	1776271	1780899	2829	942	104616.55	5.57	I	Nucleus	NIN, putative, expressed
OsNLP2	LOC_Os04g41850	Chr4	24795660	2.5E+07	2811	936	101602.95	5.72	III	Nucleus	NIN, putative, expressed
OsNLP3	LOC_Os01g13540	Chr1	7571236	7576817	2817	938	102438.72	5.66	II	Nucleus	NIN, putative, expressed
OsNLP4	LOC_Os09g37710	Chr9	21735440	21740633	2529	842	92593.99	6.29	I	Nucleus	NIN, putative, expressed
OsNLP5.1	LOC_Os11g16290	Chr11	8970074	8977435	2661	886	98080.08	6.2	III	Nucleus	Similar to RWP-RK domain containing protein, expressed
OsNLP5.2	LOC_Os11g16290	Chr11	8970074	8977435	2577	858	94889.63	6.63	III	Nucleus	Similar to RWP-RK domain containing protein, expressed
OsNLP6	LOC_Os02g04340	Chr2	1913425	1916183	2010	669	70271.09	5.17	II	Cytoplasm	NIN, putative, expressed
OsRKD1	LOC_Os01g14420	Chr1	8064796	8069095	948	315	33308.52	8.43	IV	Nucleus	RWP-RK domain-containing protein, putative, expressed
OsRKD3	LOC_Os01g37100	Chr1	20705750	2.1E+07	1326	441	47810.25	6.02	IV	Chloroplast. Nucleus	RWP-RK domain-containing protein, putative, expressed
OsRKD4	LOC_Os04g47640	Chr4	28273522	28275825	1020	339	38758.02	8.46	IV	Nucleus	RWP-RK domain-containing protein, putative, expressed
OsRKD5	LOC_Os06g12360	Chr6	6696666	6700782	2766	921	104713.13	7.36	IV	Chloroplast	RWP-RK domain-containing protein, putative, expressed
OsRKD6	LOC_Os02g51090	Chr2	31249160	31250608	699	232	27394.93	5.56	IV	Nucleus	RWP-RK domain-containing protein, putative, expressed
OsRKD8	LOC_Os12g12970	Chr12	7182492	7188162	1359	452	48036.8	5.63	IV	Nucleus	RWP-RK domain-containing protein, putative, expressed
OsRKD9	LOC_Os09g27190	Chr9	16530333	16534799	1020	339	37221.59	5.47	IV	Chloroplast. Nucleus	RWP-RK domain-containing protein, putative, expressed

Subcellular localization prediction indicated that the majority (10/14) localized exclusively to the nucleus, consistent with their predicted roles as transcription factors (**Table 1**). Notably, two exceptions were identified: OsNLP6 and OsRKD5 exhibited cytoplasmic localization (**Table 1**), suggesting potential non-nuclear functions such as post-translational regulation or signaling. Additionally, OsRKD3 and OsRKD9 showed dual cytoplasm-nucleus distribution (**Table 1**), implying shuttling dynamics that may regulate their activity.

**Figure 2:**
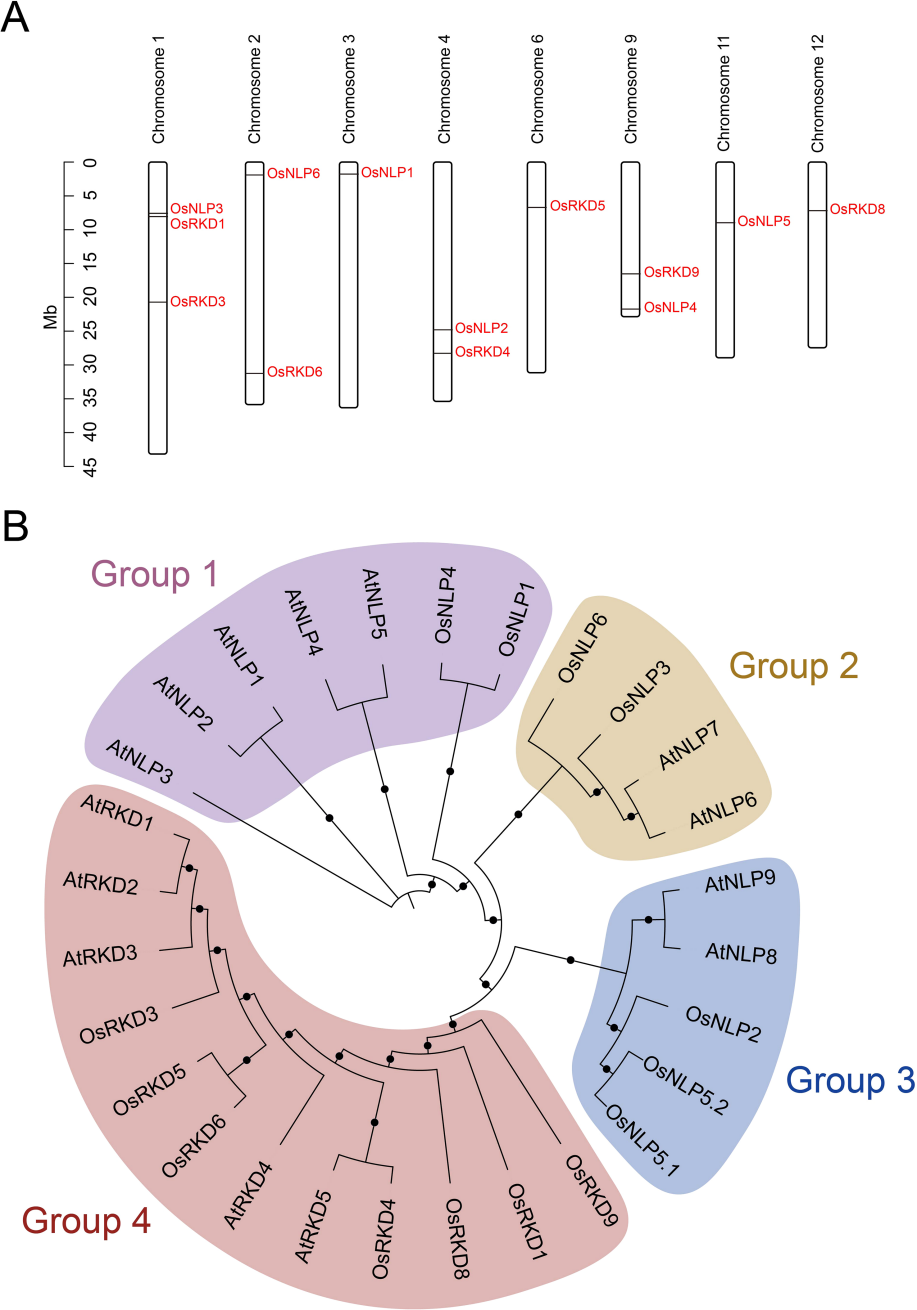
OsRWP-RK transcriptional factor family. **(A)** Chromosomal distribution of 13 OsRWP-RK genes (6 OsNLPs, 7 OsRKDs) across eight chromosomes. **(B)** Phylogenetic tree of 14 OsRWP-RK proteins, classified into four groups. Color bars denote phylogenetic groups: Group 1 (Purple), Group 2 (Brown), Group 3 (Blue), Group 4 (Red). .

These 13 OsRWP-RK genes are unevenly distributed across 8 of the 12 chromosomes, with chromosome 1 harboring three genes, chromosomes 2, 4, and 9 each containing two, and chromosomes 3, 6, 11, and 12 each with one gene (**Figure 2A**). Notably, OsNLP1, OsNLP4, and OsNLP6 are positioned near chromosome ends, suggesting potential telomeric regulatory roles (**Figure 2A**).

A phylogenetic tree of RWP-RK proteins from *Oryza sativa* and *Arabidopsis thaliana* was constructed to investigate their evolutionary relationships (**Figure 2B**). The analysis revealed that RWP-RK proteins are classified into four major groups, each containing specific genes from both species. Group 1 contains AtNLP1-5 and OsNLP1, OsNLP4. Group 2 includes AtNLP6-7, OsNLP3, and OsNLP6. Group 3 clusters AtNLP8-9, OsNLP2, OsNLP5.1, and OsNLP5.2. Group 4 contains all OsRKDs and AtRKDs. The NLP subfamily is distributed across Groups 1-3, while Group 4 represents the RKD subfamily, with clear differentiation between the two subfamilies, indicating functional divergence during evolution. The presence of multiple members in each group suggests that gene duplication events contributed to the expansion of the gene family. Furthermore, the clustering of genes from both *Arabidopsis* and rice indicates a shared evolutionary origin, suggesting that the RWP-RK gene family is ancient and conserved across species.

### 3.2 Gene structures, conserved motifs, and protein domains

To elucidate structural diversity among OsRWP-RK genes, we analyzed their exon-intron organization, conserved motifs, and protein domain (**Figures 1A, B**). The genes consist of 3-6 introns and 4-7 exons, exhibiting variation in exon/intron length and arrangement. Additionally, structural differences between the two subfamilies may result in distinct regulatory patterns or expression profiles, potentially influencing their biological functions.

Motifs are small, conserved sequence fragments with functional significance in proteins. Motif analysis helps determine protein function. Using the MEME tool, three conserved motifs were identified in OsRWP-RK proteins (**Figure 1C**). Each protein contained 1-5 conserved motifs. The RKD subfamily retained the same functional motifs, while the NLP subfamily acquired two additional motifs, reflecting functional differences between the subfamilies.

Protein conserved domain analysis reveals core functional regions, evolutionary history, and biological functions of proteins. In this study, conserved domains were identified using the NCBI CDD database (**Figure 1D**). The results demonstrated that OsRKD subfamily members contain a single RWP-RK domain. In contrast, OsNLP proteins possess both the RWP-RK and PB1 domains (**Figure 1D**). This suggests that NLP subfamily members may adapt to specific functions by acquiring new domains, while RKD members retain their original domains to maintain basic functions. Additionally, the PB1 domain in the OsNLP subfamily is predominantly located at the C-terminus (**Figure 1D**), and the conserved spatial relationship between the RWP-RK and PB1 domains suggests a shared origin. Notably, OsRKD5 contains both a PLN03077 and a GBP_C domain (**Figure 1D**), indicating that the gene acquired new functions during evolution, enhancing rice’s adaptation to its environment.

### 3.3 Protein structure and evolutionary features

**Figure 3:**
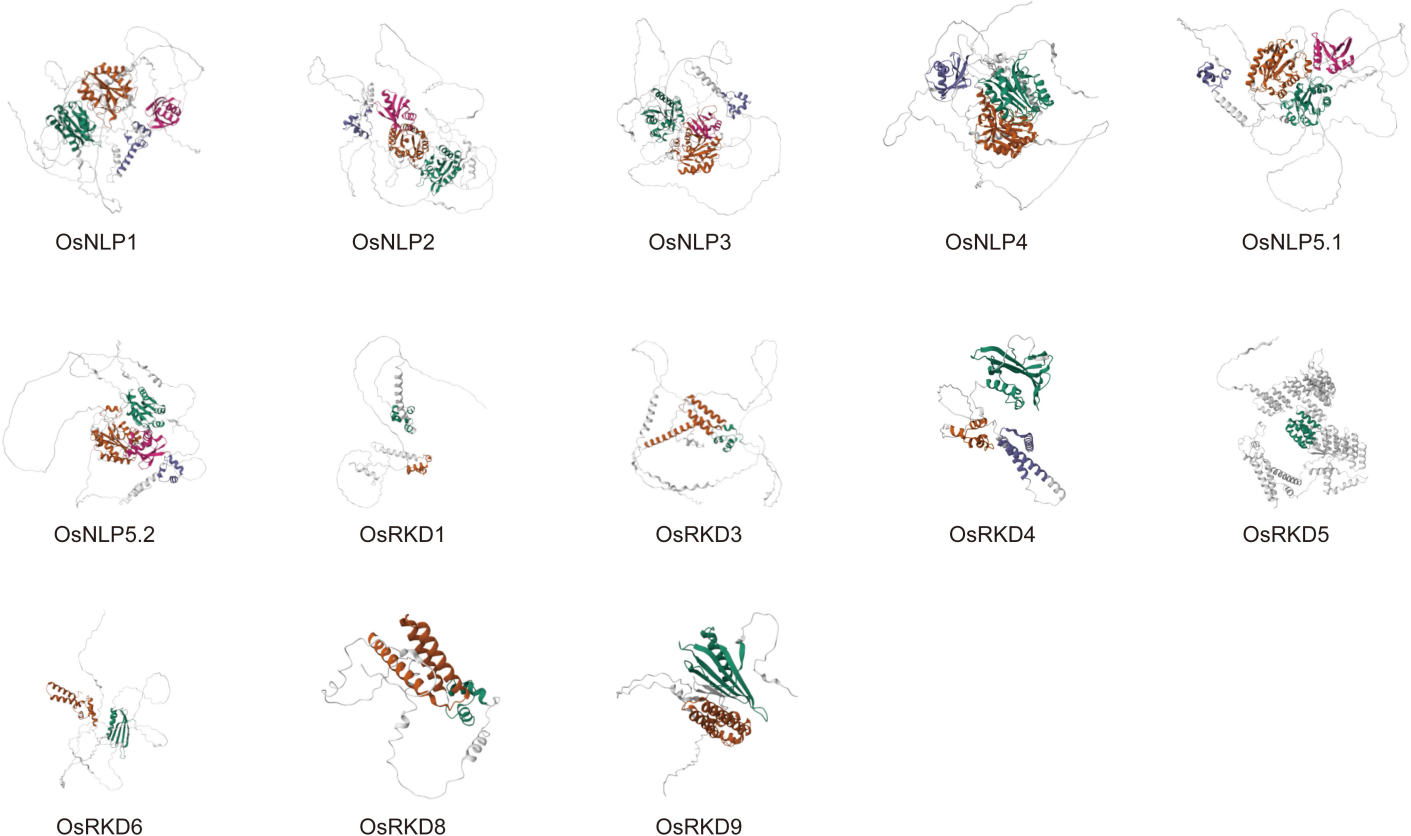
Predicted 3D structures of OsRWP-RK proteins. Helices are represented as spirals, β-pleated sheets as broad strips, and coils as thin loops. Each protein contains up to four domains, labeled from the N-terminus to the C-terminus in green, orange, purple, and pink, respectively.

The 3D structure of a protein dictates its function, with features such as the catalytic site and ligand binding site determined by its spatial arrangement. We predicted the 3D structures of OsRWP-RK family proteins, including OsNLP and OsRKD subfamily members (**Figure 3**; **Supplementary Figure 2**). The models reveal differences in domain composition and overall folding. The OsNLP subfamily exhibits a relatively conserved 3D structure. Two large conserved domains (green and orange) are consistently located at the N-terminus (**Figure 3**), forming the structural core and occupying a substantial volume. Two smaller regions, located near the C-terminus (with OsNLP4 containing only one), are spatially arranged around the large regions (**Figure 3**). These domains are connected by flexible loops, suggesting potential cooperative function. Based on **Figure 1D**, the two small C-terminal domains are likely the RWP-RK domain (purple) and the PB1 domain (pink). Their spatial positioning suggests favorable orientation for DNA binding and nitrate signal perception (Konishi and Yanagisawa, 2013, 2019). In contrast, OsRKD proteins display greater structural variability, indicating potential functional diversity. Most OsRKD members exhibit a simplified architecture, often retaining only a single RWP-RK domain. This minimal structure may be sufficient for specific transcriptional regulation, such as the activation of genes involved in reproduction (Purwestri et al., 2023). Notably, OsRKD5 possesses a more complex structure, potentially due to the presence of additional domains—PLN03077 and GBP_C—as shown in **Figure 1D**. These structural characteristics provide valuable insights into the functional specialization and regulatory mechanisms of the OsRWP-RK protein family.

**Figure 4:**
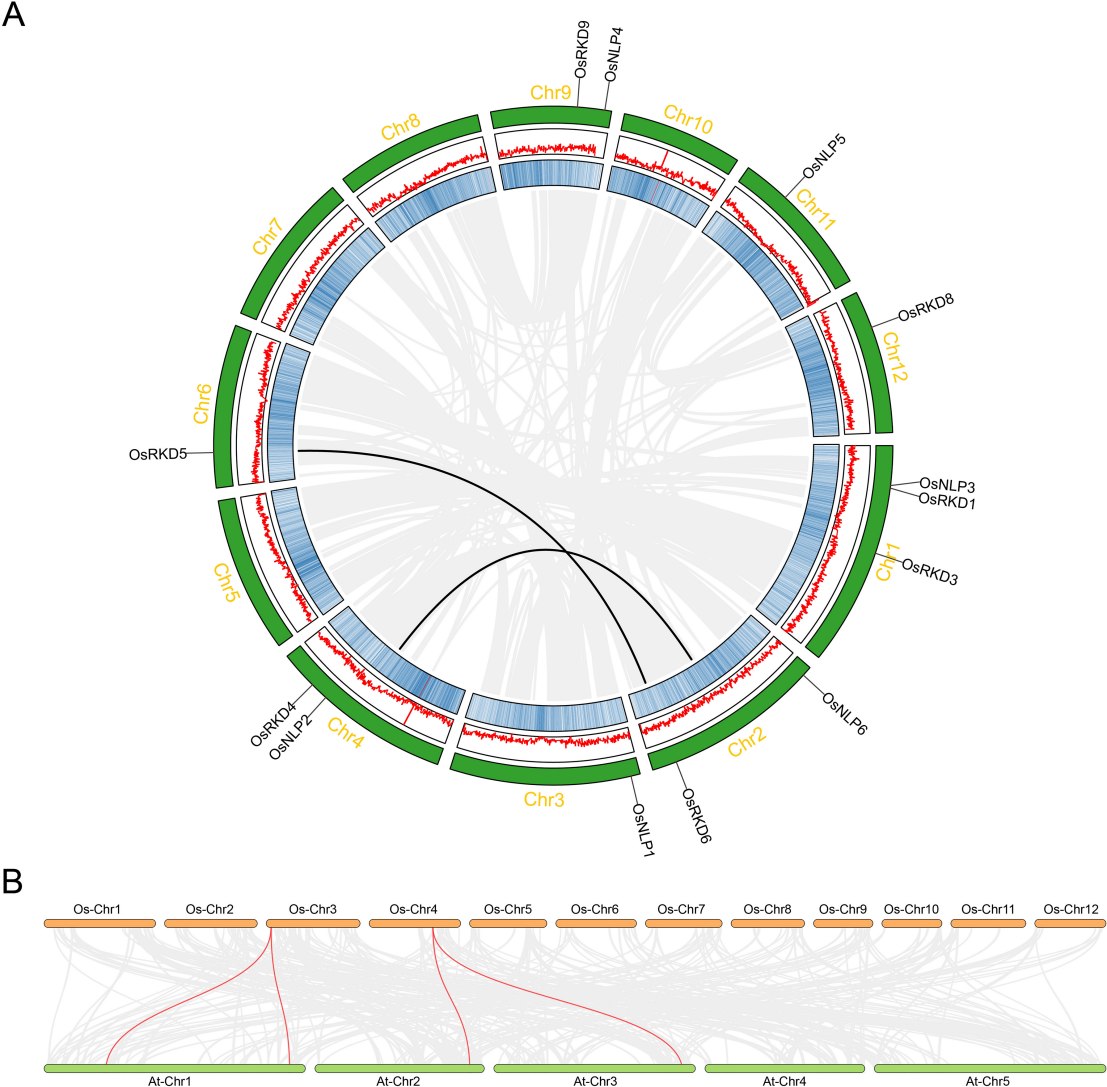
Collinearity and synteny analysis of OsRWP-RK genes. **(A)** Collinearity analysis of OsRWP-RK genes in *Oryza sativa*. Gray lines inside the circle represent all collinear gene pairs within the rice genome, while red lines highlight segmental duplication events within the OsRWP-RK family. Chromosome numbers are labeled outside the green boxes. Gene density is depicted using a blue heatmap in the inner boxes and red lines in the middle boxes. The chromosomal locations of OsRWP-RK genes are marked by black lines. **(B)** Synteny analysis between *Oryza sativa* and *Arabidopsis thaliana*. Gray lines indicate all collinear gene pairs between the two species, whereas red lines highlight collinear OsRWP-RK gene pairs.

Synteny analysis of gene families reveals their evolutionary history, functional conservation, and inter-species relationships, and provides insights into adaptation, environmental response, and functional evolution. In this study, synteny analysis of the OsRWP-RK family highlights the conservation of the RWP-RK genes in rice and *Arabidopsis*, shedding light on their evolutionary patterns (**Figure 4**). The analysis shows significant collinearity within the rice genome, such as between OsRKD6 (Chromosome 6) and OsRKD5 (Chromosome 2) (**Figure 4A**). Additionally, OsNLP2 (Chromosome 4) exhibits strong collinearity with a region on chromosome 2 (**Figure 4A**), suggesting gene duplication and evolutionary divergence, leading to the distribution and functional diversification of the OsRWP-RK family in rice. Furthermore, synteny between the rice and *Arabidopsis* RWP-RK gene families reveals four collinear gene pairs (**Figure 4B**), indicating that these families have retained conservation through evolution, likely originating from a common ancestor and undergoing gene recombination and species-specific divergence over time.

### 3.4 Analysis of OsRWP-RK gene promoters

**Figure 5:**
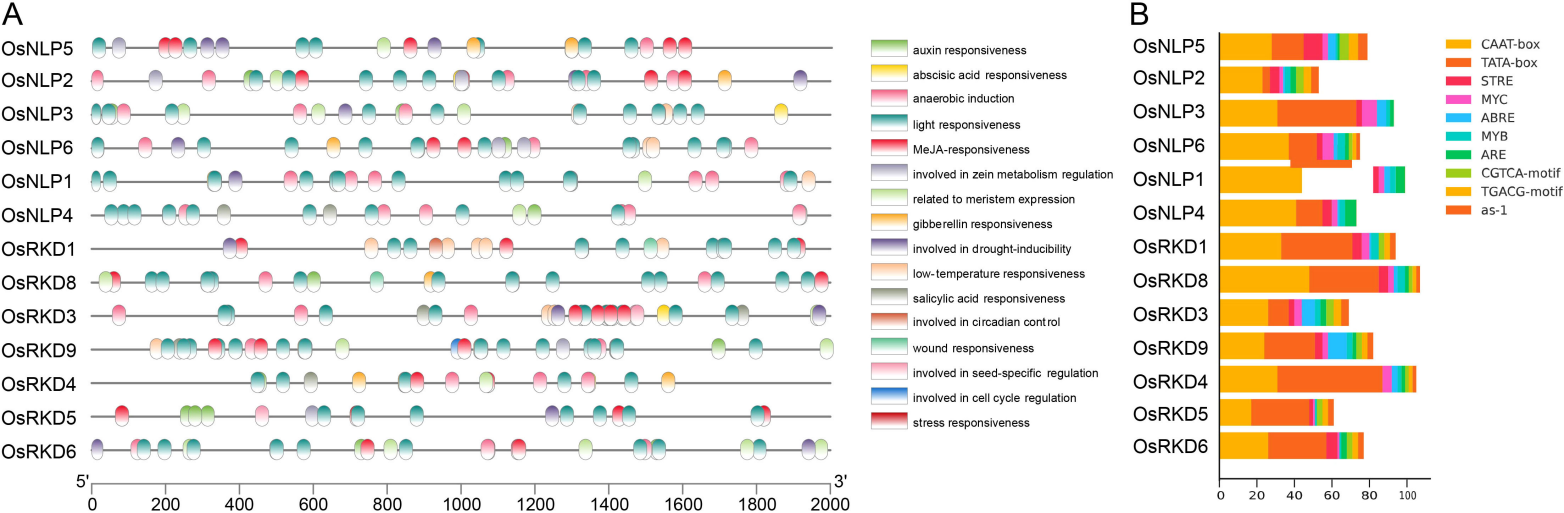
Cis-element analysis in OsRWP-RK gene promoters. **(A)** Distribution of cis-elements in OsRWP-RK gene promoters, with each element represented by a distinct color. **(B)** Top 10 cis-element count for each OsRWP-RK gene.

Cis-acting elements are crucial for gene regulation and can predict gene expression patterns under different tissue, organ, or environmental conditions. In this study, promoter analysis revealed diverse cis-regulatory elements in the 2 kb upstream regions of OsRWP-RK genes. Among the 80 detected elements, the CAAT-box and TATA-box are the most critical elements (**Figure 5B**; **Supplementary Data Sheet S2**). The CAAT-box enhances basal transcription by binding to CAAT-binding proteins, while the TATA-box facilitates the assembly of transcription initiation complexes, aiding RNA polymerase II in locating the transcription start site. Abundant cis-elements associated with hormone and abiotic stress responses were also identified (**Figure 5A**). Notable hormone-related cis-elements include ABRE (abscisic acid response), TGACG-motif and CGTCA-motif (jasmonic acid response), and as-1 (salicylic acid response). Abiotic stress-related elements include MYB and G-box (light response), MYC and MBS (drought response), MYC and LTR (low-temperature response), ARE (anaerobic response), and STRE (stress-responsive) (**Figures 5A, B**; **Supplementary Data Sheet S2**). All in all, the promoter region of the OsRWP-RK family contains various cis-acting elements that regulate gene expression and are involved in hormonal and stress responses.

### 3.5 Transcription profiles of OsRWP-RK genes in different tissues

**Figure 6:**
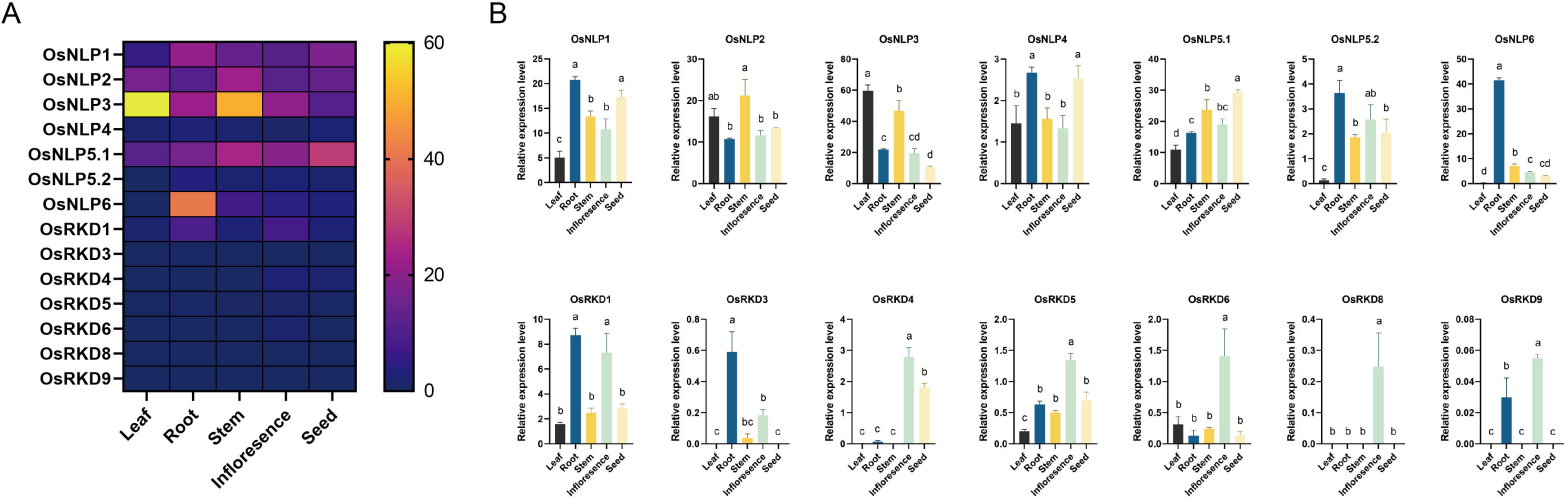
Expression patterns of OsRWP-RK genes in *Oryza sativa*. **(A)** Heatmap showing the expression levels of all OsRWP-RK genes across different tissues. **(B)** Bar graph depicting the expression levels of individual OsRWP-RK genes. Different letters indicate statistically significant differences between tissues (p < 0.05).

To investigate the transcription patterns of OsRWP-RK genes, we obtained a gene expression matrix from the Rice Gene Annotation Project (https://rice.uga.edu/expression.shtml; accessed on 7 Jan 2025) (**Figure 6**). Most NLPs are expressed across all tissues, suggesting their role in growth regulation in rice. (**Figures 6A, B**). OsNLP1-3, OsNLP5.1, and OsNLP6 are expressed at significantly higher levels in roots (TPM>10; **Figures 6A, B**), suggesting shared transcriptional regulation under variational nutrient conditions. Furthermore, the OsRKD subfamily is generally expressed at low levels across tissues (TPM<10; **Figure 6A**), while OsRKD1, OsRKD4, OsRKD6, OsRKD8, and OsRKD9 show relatively higher expression in inflorescences, exceeding levels in other tissues by at least two-fold (**Figures 6A, B**), further supporting subfamily-specific functional roles.

### 3.6 Nitrogen-induced expression dynamics of OsRWP-RK genes

**Figure 7:**
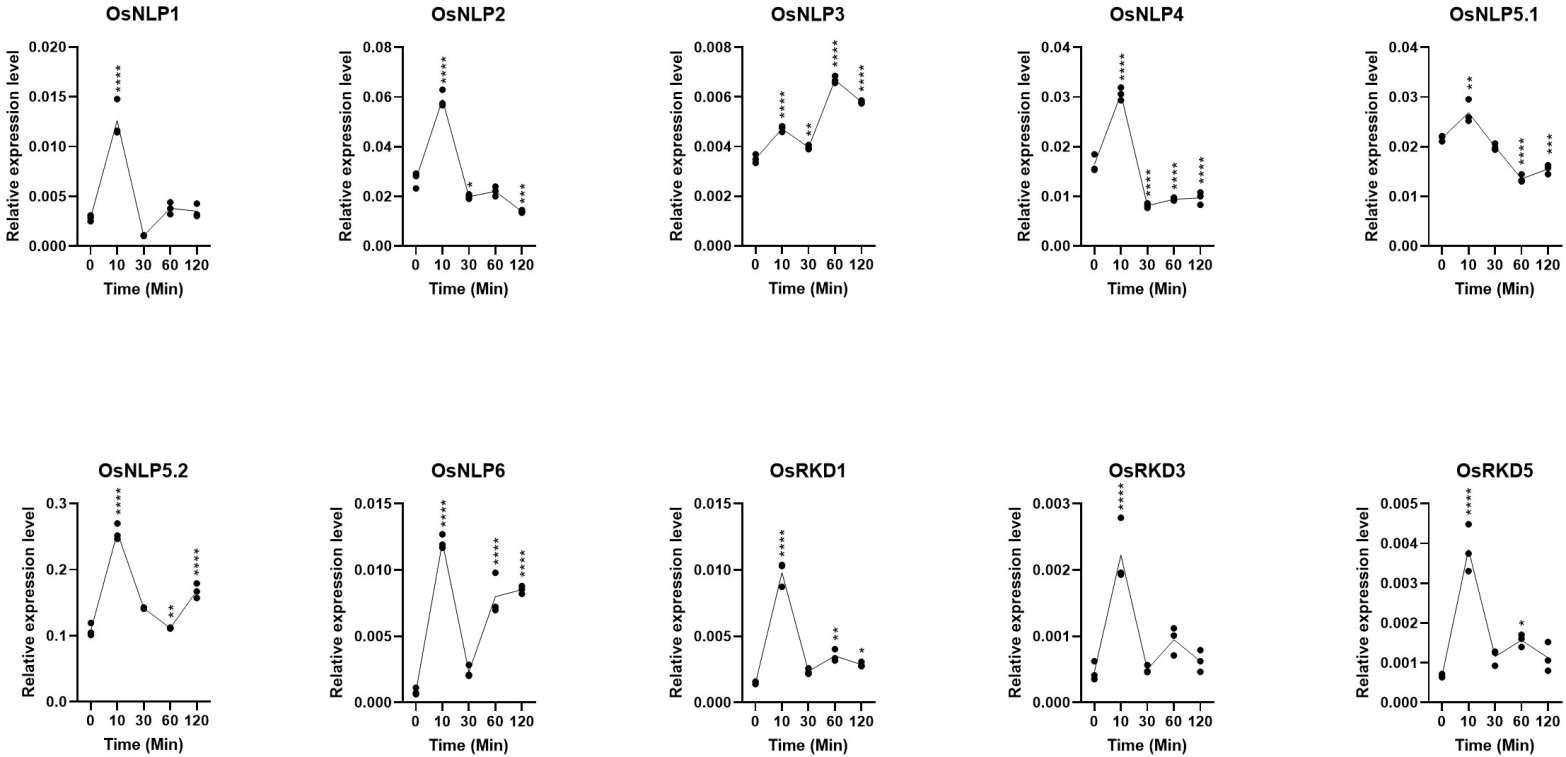
Fluctuations in OsRWP-RK gene expression after nitrogen-starved seedlings were reintroduced to a nitrogen-contain environment. (*p ≤ 0.05; **p ≤ 0.01; ***p ≤ 0.001, ****p ≤ 0.0001).

To further explore the diversity of transcriptional responses among OsRWP-RK genes upon nitrogen re-supply, we analyzed temporal expression profiles of 10 representative genes using qRT-PCR across five time points (0, 10, 30, 60, and 120 minutes; **Figure 7**). Most genes responded rapidly to nitrogen, with peak expression occurring at 10 minutes. For example, OsNLP1 and OsRKD3 showed strong and transient upregulation, followed by a sharp decline to near-baseline levels by 30 minutes. These early-transient responders may act as immediate nitrogen sensors or signal initiators. OsNLP2, OsNLP4, and OsNLP5.1 were also initially induced at 10 minutes, but their transcript levels subsequently declined to below or near baseline. This pattern suggests a transient and tightly controlled regulatory role, potentially functioning in early signaling checkpoints. OsNLP5.2 and OsRKD1 maintained elevated expression levels throughout the time course, indicating prolonged involvement in transcriptional regulation during the nitrogen response. OsNLP6 exhibited a unique two-phase expression pattern, with an initial increase at 10 minutes, a transient decrease at 30 minutes, and a second rise at 60 minutes. This oscillatory behavior may reflect feedback regulation or involvement in multiple signaling phases. OsNLP3 displayed a gradual increase in expression, reaching peak levels at 60 minutes. This delayed response suggests a role downstream of initial nitrogen signaling, possibly in the regulation of secondary targets (Zhang et al., 2022).

## 4 Discussion

The discovery and functional analysis of RWP-RK proteins revolutionized our understanding of nitrogen signaling and gametophyte development across plants (Marchive et al., 2013; Chardin et al., 2014; Tedeschi et al., 2017). Rice, as a major cereal crop, represents an exemplary model system for dissecting these processes. Although previous studies reported 15 RWP-RK genes in rice (Zheng et al., 2024), our analysis revealed that the proteins of OsRKD7 (*LOC_Os08g19820*) and OsRKD10 (*LOC_Os02g20530*) in the Nipponbare cultivar lack RWP-RK domains. Thus, 13 OsRWP-RK genes were identified in this study (**Table 1**) and unevenly distributed across eight of the 12 chromosomes (**Figure 2A**). Additionally, 37 RWP-RK genes were identified in wheat (Kumar et al., 2018) and 12 ZmNLP genes in maize (Ge et al., 2018). Compared to wheat and maize, the RWP-RK gene family in *Oryza sativa* underwent a conservative expansion. Moreover, NLP subfamily proteins generally have longer CDS and higher molecular weights (**Table 1**; **Figure 1**), similar to those in wheat and maize (Ge et al., 2018; Kumar et al., 2018).

The RWP-RK transcription factor family is divided into two subfamilies: RKDs and NLPs. RKDs contain a conserved RWP-RK domain (Yin et al., 2020). NLPs, in contrast, possess both RWP-RK and PB1 domains, along with a GAF-like structure domain (Schauser et al., 2005). All OsNLPs possess PB1 domains—facilitating protein–protein interactions—and RWP-RK domains, indicating roles in transcriptional complexes responsive to nitrogen (**Figure 1D**). In contrast, RKDs retain only the RWP-RK domain, possibly reflecting specialized roles in gametophyte development (**Figure 1D**). OsRKD5 uniquely contains PLN03077 and GBP_C domains, suggesting an evolutionary innovation that may broaden its regulatory scope (**Figure 1D**).

Gene duplication is a key driver of gene functional diversification and species evolution (Bowers et al., 2003). In eukaryotes, duplication events occur primarily through tandem or segmental duplication (Cannon et al., 2004). Studies suggest that the NLP subfamily in *Arabidopsis* evolved via segmental rather than tandem duplication (Schauser et al., 2005). In this study, we identified two segmentally duplicated gene pairs (**Figure 4A**), suggesting that segmental duplication also contributed to the expansion of OsNLP genes. Synteny analysis of the rice and *Arabidopsis* RWP-RK gene families reveals four collinear gene pairs (**Figure 4B**), suggesting they share a common ancestor and have conserved through evolution.

Cis-acting elements are crucial for gene regulation and can predict gene expression patterns under different tissue, organ, or environmental conditions. We identified 80 types of cis-acting elements in OsRWP-RK promoters (**Supplementary Data Sheet S2**), which may contribute to the differential expression of RWP-RK genes across tissues. However, some genes with diverse cis-acting elements exhibited consistently low expression in all tested tissues (**Figures 5**, **6**), suggesting that gene expression is influenced by additional factors beyond promoter elements. Epigenetic modifications, such as DNA methylation, may contribute to this low expression (Zhang et al., 2018). Furthermore, an analysis combining gene expression patterns and promoter cis-element composition revealed that most genes showed higher expression in leaves and roots (**Figures 5**, **6A**), potentially due to the abundance of light-responsive elements and stress-responsive OsRWP-RK promoters (**Figure 5**). Additionally, the presence of numerous hormone-responsive elements (**Figure 5**) raises the intriguing possibility that these elements mediate crosstalk between the RWP-RK gene family and hormone signaling pathways, warranting further investigation.

In *Oryza sativa*, 13 OsRWP-RK genes (14 transcripts) were identified across leaves, roots, stems, inflorescences, and seeds. OsNLP genes exhibited broad expression, particularly in roots, stems, and leaves (**Figure 6**), supporting a broad regulatory role in various physiological processes, such as nitrogen uptake, transport, and redistribution to regulate plant growth and development (Liu et al., 2018). In contrast, most OsRKD members are expressed higher in inflorescences than elsewhere, exceeding other tissues by at least twofold. Previous research has found that OsRKD3 in Indonesian black rice (Cempo Ireng), which is resistant to somatic embryogenesis, can induce somatic embryo formation (Purwestri et al., 2023). Similarly, the RKD-type RWP-RK transcription factor Shohai1 is essential for embryo and endosperm development in maize (Mimura et al., 2018). This indicated that RKDs contribute to gametophyte development.

Furthermore, the expression levels of all OsNLP genes and OsRKD1, OsRKD3, and OsRKD5 fluctuated upon transferring nitrogen-starved seedlings to a nitrogen-sufficient environment. This suggests that OsRWP-RK genes play pivotal roles in nitrate response and nitrogen starvation signaling. Given the evolutionary expansion and sub-functionalization of the OsRWP-RK subfamily, these genes likely employ diverse molecular mechanisms to respond to external nitrogen fluctuations.

In summary, RWP-RK genes in *Oryza sativa* show diverse expression patterns, with OsNLP genes primarily expressed in nutritive organs and OsRKD genes involved in gametogenesis and embryogenesis. This study offers new insights into the role of RWP-RKs in seed development and nitrogen response.

## 5 Conclusion

In this study, we performed a genome-wide identification of the RWP-RK family in *Oryza sativa*, analyzed their expression across five tissues, and examined their transcriptional responses to nitrogen availability. A total of 13 RWP-RK genes were identified, including OsNLP5, which encodes two proteins containing RWP-RK domains. These genes are classified into two subfamilies—OsNLP (six genes) and OsRKD (seven genes)—distributed across eight of the 12 rice chromosomes.

Significant structural differences exist between the two subfamilies: OsNLP genes are longer in gene and coding sequence (CDS) length, protein size, and molecular weight, with more complex 3D structures than OsRKD genes. While OsRKD members retain a single RWP-RK domain, OsNLP members possess both RWP-RK and PB1 domains, suggesting functional specialization. Promoter analysis revealed diverse cis-acting elements associated with hormone and stress responses.

Expression analysis indicated that OsNLP genes exhibited broad expression, supporting a broad regulatory role in various physiological processes. In contrast, OsRKD genes generally showed low expression but were highly expressed in inflorescences, implying involvement in gametophyte development. Additionally, all NLP genes, along with OsRKD1, OsRKD3, and OsRKD5, exhibited similar overall expression fluctuations with subtle differences upon nitrogen reintroduction, suggesting diverse molecular responses to nitrate availability.

In summary, this genome-wide characterization of RWP-RK transcription factors in *Oryza sativa* provides a theoretical framework for future research on the regulatory mechanisms of germ cell differentiation and embryo development. Moreover, this study could contribute to the development of rice varieties with improved nitrogen use efficiency, which is crucial for sustainable agriculture.

## Data Availability

The original contributions presented in the study are included in the article/**Supplementary Material**. Further inquiries can be directed to the corresponding author.

## Supplementary material

Supplementary Figure 1 The differences in gene and protein structures between the OsNLP and OsRKD subfamilies. The OsNLP subfamily is larger than the OsRKD subfamily in CDS length **(A)**, protein length **(B)**, protein molecular weight **(C)**, and protein domain number **(D)**.

Supplementary Figure 2 Predicted 3D structures of OsRWP-RK proteins. Helices are represented as spirals, β-pleated sheets as broad strips, and coils as thin loops. AlphaFold produces a per-residue model confidence score (pLDDT) between 0 and 100. Some regions below 50 pLDDT may be unstructured in isolation (dark blue, pLDDT > 90, light blue, 90 > pLDDT > 70, yellow, 70 > pLDDT > 50, orange, pLDDT < 50).
