# Supplementary figures and images for: Genome-wide analysis of RWP-RK transcription factor family reveals its roles in nitrogen response in rice (Oryza sativa)
Source: Front Plant Sci. 2025 Sep 8;16:1597029. doi: 10.3389/fpls.2025.1597029 (PMC12450915; doi:10.3389/fpls.2025.1597029)

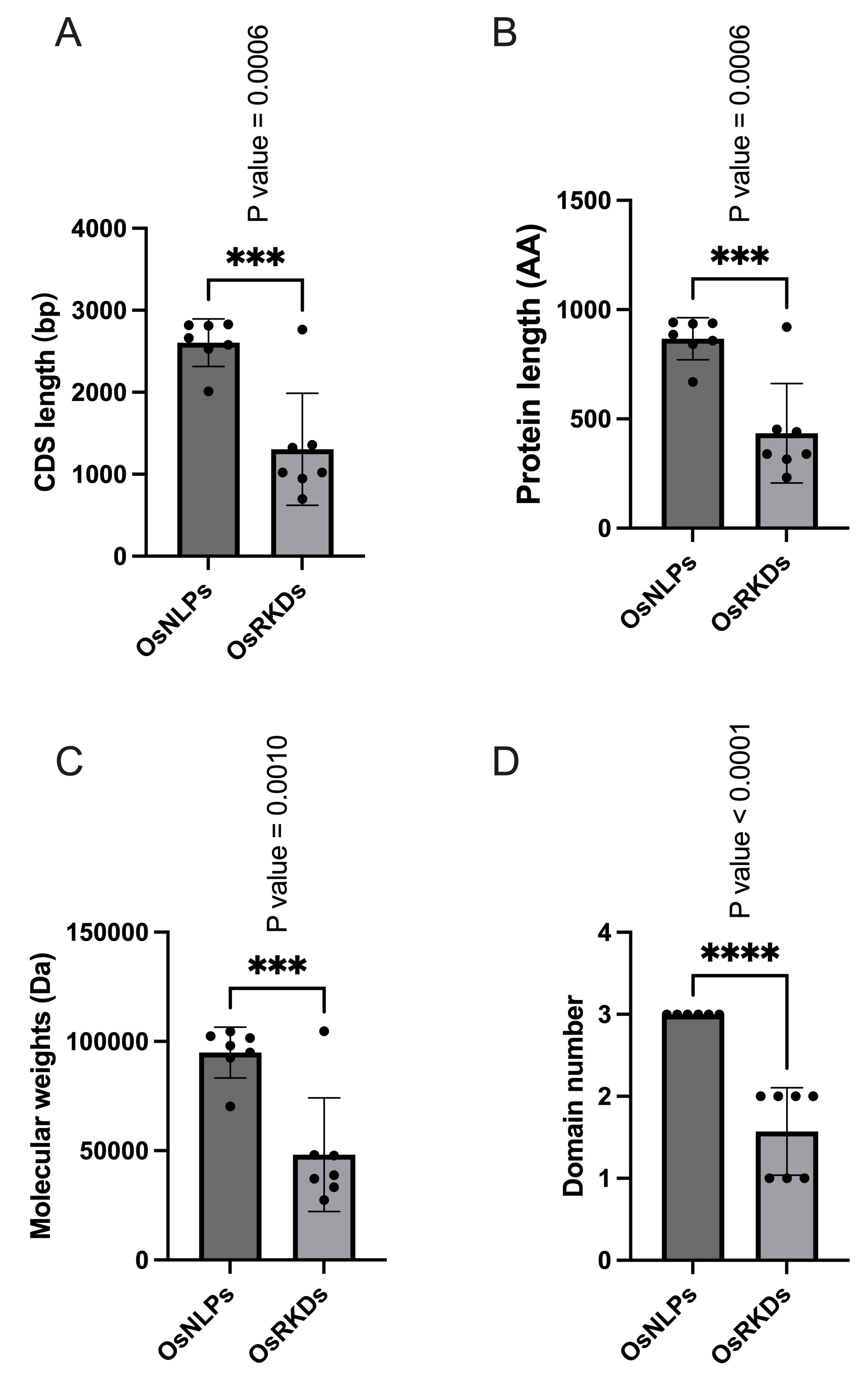

Supplement: Supplementary Figure 1 — The differences in gene and protein structures between the OsNLP and OsRKD subfamilies. The OsNLP subfamily is larger than the OsRKD subfamily in CDS length (A), protein length (B), protein molecular weight (C), and protein domain number (D). [file Image1.jpeg]

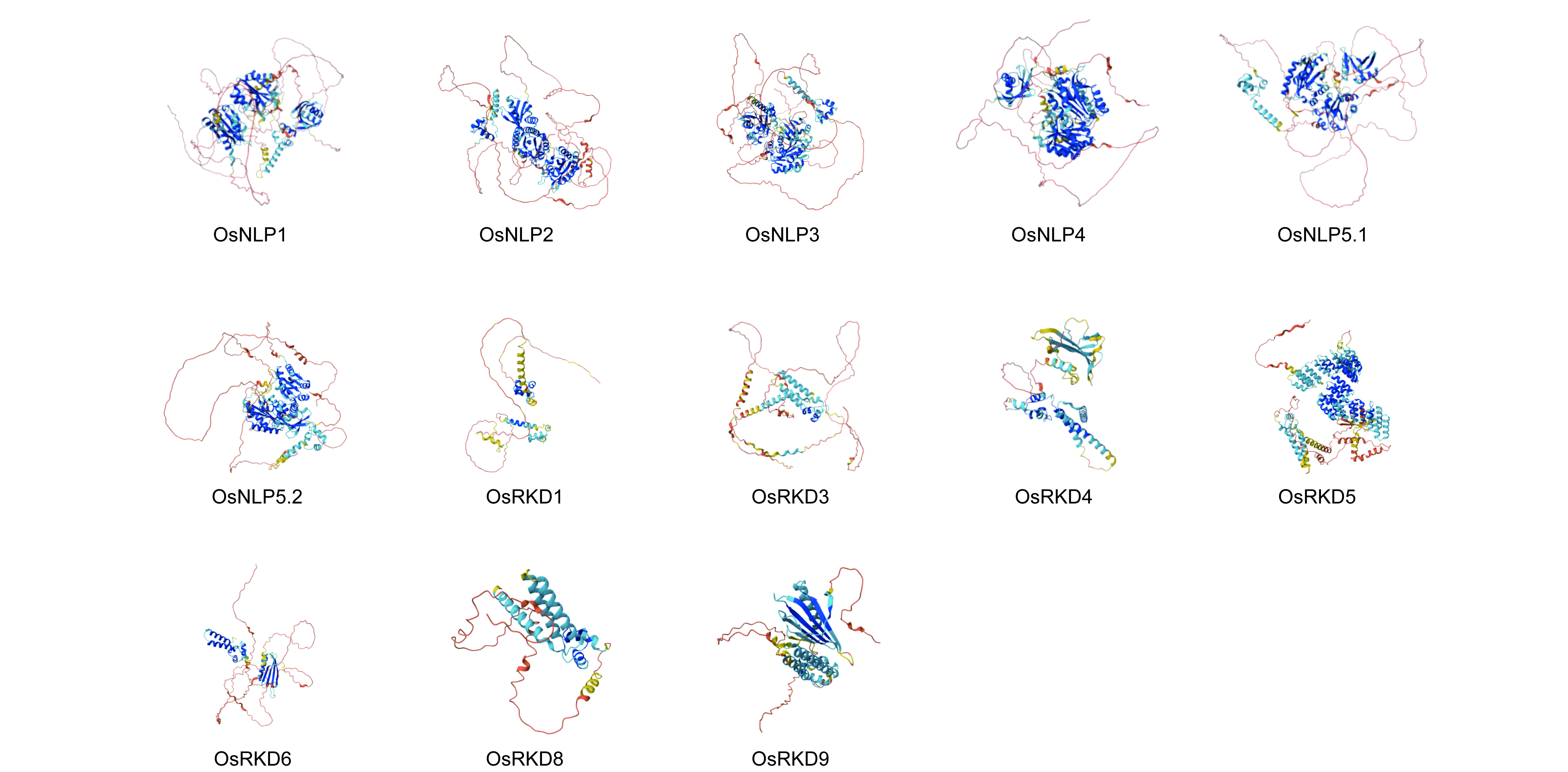

Supplement: Supplementary Figure 2 — Predicted 3D structures of OsRWP-RK proteins. Helices are represented as spirals, β-pleated sheets as broad strips, and coils as thin loops. AlphaFold produces a per-residue model confidence score (pLDDT) between 0 and 100. Some regions below 50 pLDDT may be unstructured in isolation (dark blue, pLDDT > 90, light blue, 90 > pLDDT > 70, yellow, 70 > pLDDT > 50, orange, pLDDT < 50). [file Image2.jpg]
